# Reactivation of Deep Subsurface Microbial Community in Response to Methane or Methanol Amendment

**DOI:** 10.3389/fmicb.2017.00431

**Published:** 2017-03-17

**Authors:** Pauliina Rajala, Malin Bomberg

**Affiliations:** ^1^Materials Performance, VTT Technical Research Centre of FinlandEspoo, Finland; ^2^Material Processing and Geotechnology, VTT Technical Research Centre of FinlandEspoo, Finland

**Keywords:** deep biosphere, deep life, carbon, methane, methanol, methanotrophy

## Abstract

Microbial communities in deep subsurface environments comprise a large portion of Earth’s biomass, but the microbial activity in these habitats is largely unknown. Here, we studied how microorganisms from two isolated groundwater fractures at 180 and 500 m depths of the Outokumpu Deep Drillhole (Finland) responded to methane or methanol amendment, in the presence or absence of sulfate as an additional electron acceptor. Methane is a plausible intermediate in the deep subsurface carbon cycle, and electron acceptors such as sulfate are critical components for oxidation processes. In fact, the majority of the available carbon in the Outokumpu deep biosphere is present as methane. Methanol is an intermediate of methane oxidation, but may also be produced through degradation of organic matter. The fracture fluid samples were incubated *in vitro* with methane or methanol in the presence or absence of sulfate as electron acceptor. The metabolic response of microbial communities was measured by staining the microbial cells with fluorescent redox sensitive dye combined with flow cytometry, and DNA or cDNA-derived amplicon sequencing. The microbial community of the fracture zone at the 180 m depth was originally considerably more respiratory active and 10-fold more numerous (10^5^ cells ml^-1^ at 180 m depth and 10^4^ cells ml^-1^ at 500 m depth) than the community of the fracture zone at the 500 m. However, the dormant microbial community at the 500 m depth rapidly reactivated their transcription and respiration systems in the presence of methane or methanol, whereas in the shallower fracture zone only a small sub-population was able to utilize the newly available carbon source. In addition, the composition of substrate activated microbial communities differed at both depths from original microbial communities. The results demonstrate that OTUs representing minor groups of the total microbial communities play an important role when microbial communities face changes in environmental conditions.

## Introduction

The Fennoscandian Shield, i.e., the exposed Precambrian northwest segment of the East European Craton, preserves some of the oldest geological records of Earth. Studies conducted in several terrestrial deep sites have reported diverse microbial communities extending several kilometers into the Earth’s crystalline bedrock (Pedersen, 1997; Haveman and Pedersen, 1999; Sass and Cypionka, 2004; Wu et al., 2015). Microbial communities in deep subsurface environments have been estimated to comprise a large portion of Earth’s biomass (McMahon and Parnell, 2014) and to have a great impact on the elemental cycles in the deep biosphere (Gadd, 2010). The deep groundwater in crystalline continental crust, such as the one of the Fennoscandian Shield, is independent of photosynthetic carbon and energy production. Alternative means of carbon assimilation, such as inorganic carbon fixation or methane oxidation, may be important to support the microbial communities in these conditions. Chemolithoautotrophic organisms are thought to be the primary producers in deep crystalline bedrock environments (Pedersen, 1997, 2000).

Microorganisms in the deep, nutrient limited biosphere are believed to conduct only little metabolic activity due to low nutrient availability in these environments (Jørgensen, 2011; Hoehler and Jørgensen, 2013). Dormancy refers to an ability to enter a reversible state of low metabolic activity under unfavorable environmental conditions. By remaining in dormant state the microorganisms lower their energetic expenditures. The dormant microorganisms generate a seed bank that may activate following beneficial environmental change (Lennon and Jones, 2011). However, dormant microorganisms must invest resources into resting structures and the machinery that is needed for transitioning into and out of a dormant state (Lennon and Jones, 2011). In addition, low amount of maintenance and survival energy is still required ensure the long-term viability of dormant microorganisms (van Bodegom, 2007; Hoehler and Jørgensen, 2013).

The Outokumpu Deep Drillhole, located in eastern Finland, provides access to study isolated aquifers in the crystalline bedrock in the Paleoproterozoic part of the Fennoscandian Shield (Kukkonen, 2011). The bedrock in Outokumpu is composed of several geochemically different rock types. These include metasediments, ophiolite-derived altered ultramafic rocks and pegmatitic granite (Ahonen et al., 2011). The drillhole reaches a total depth of 2516 m and spans several fluid-filled fracture zones. Formation waters in these fracture zones are old, highly saline, reducing and have low organic carbon content. The dissolved gas phase, of which CH_4_ covers up to 80 vol-%, is abundant (Kietäväinen et al., 2013). The fracture waters have been categorized to five water types, type I (180 m), type II (500–967 m), type III (1500 m), type IV (1820–2260 m) and type V (2300 m) (Kietäväinen et al., 2013). The type I water has characteristically high pH (around 10) and higher alkalinity than the other water types in Outokumpu. High pH in the drillhole water column may originate from concrete casing within the uppermost 30 m of the drillhole, and during long-term purging of the sealed-off 180 m fracture zone, the pH of the native aquifer fluid dropped to a stable level of 8.5. Water type II contains the highest amount of dissolved gases in the whole water column, of which approximately 75% (22–32 mmol l^-1^) is methane. A diverse bacterial and archaeal community has been detected down to 2300 m depth in the drillhole (Purkamo et al., 2013, 2015, 2016; Nyyssönen et al., 2014). These communities vary at different sampling depths in response to prevailing lithology and hydrogeochemistry (Purkamo et al., 2014, 2016). Simple one-carbon compounds, such as methane and methanol are important intermediates in the deep subsurface carbon cycle, and electron acceptors such as sulfate are critical components in the oxidation processes (Kietäväinen and Purkamo, 2015). Methane (CH_4_) is a key compound in the global carbon cycle and a dominant gas in many Precambrian continental bedrock formations (reviewed by Kietäväinen and Purkamo, 2015). In the shallow subsurface CH_4_ is mainly produced by anaerobic digestion of organic matter. Deeper in the geological strata CH_4_ is found in large quantities within sedimentary formations (Arthur and Cole, 2014). Many aspects of origin, source, and cycling of CH_4_ in deep continental bedrock environments still remain poorly understood (Kietäväinen and Purkamo, 2015). The study of methane cycling is important in order to understand deep subsurface ecosystems, such as those of the Outokumpu deep subsurface. The microbial communities in the Outokumpu deep subsurface have earlier been demonstrated to contain genes involved in methane cycling, indicating both methanogenesis and methanotrophy (Nyyssönen et al., 2014; Purkamo et al., 2015; Rajala et al., 2015). Methanol may be produced through degradation of recalcitrant organic compounds present in, e.g., black schist (Petsch, 2001). Additionally, Nyyssönen et al. (2014) suggested possibility of methylotrophic methanogenesis on Outokumpu deep drillhole water column.

The aim of present work was to determine the activity of microbial communities of two isolated bedrock fractures at 180 and 500 m depths of the Outokumpu deep subsurface and ability of the dormant fraction of these microbial communities to re-activate. Methane or methanol amendments were used as activating substances in the presence or absence of sulfate as an electron acceptor. The fracture fluid samples were incubated *in vitro* with methane or methanol in the presence or absence of sulfate as electron acceptor. High-throughput amplicon sequencing of bacterial and archaeal 16S rRNA genes and gene transcripts were used to identify and characterize the microorganisms that rapidly activate their metabolism in response to methane or methanol. Metabolic response was measured also by staining the microbial cells with fluorescent dyes that indicate respiratory activity. One of the main objectives of this study was thus to determine the microbial community activity and identity of microorganisms responding to methane or methanol in Outokumpu deep biosphere.

## Materials and Methods

### Description of the Outokumpu Deep Drillhole

The Outokumpu deep drillhole is situated in Outokumpu, eastern Finland (62°43′ 04′′ N, 29°3′ 43′′ E), in a Paleoproterozoic sequence consisting of metasediments, ophiolite-derived altered ultramafic rocks and pegmatitic granite (Ahonen et al., 2011). The Outokumpu mica gneiss- and granite-dominated rock association represents typical Fennoscandian bedrock. A 22 cm wide drillhole was drilled in 2004–2005 to a total depth of 2516 m and spans several independent bedrock fracture zones (Kukkonen, 2011; Kietäväinen et al., 2013) through Paleoproterozoic, approximately 2 Ga old, bedrock. The lithology, hydrogeochemistry and gas composition of the drillhole and fracture zones have been described previously (Ahonen et al., 2011; Kietäväinen et al., 2013; Purkamo et al., 2013; Nyyssönen et al., 2014). The fracture zones at 180 and 500 m depth are situated in a metasedimentary rock sequence, predominated by mica schist/biotite gneiss (Purkamo et al., 2014). The residence time for the fracture waters have been estimated to be between 20 and 50 Ma (Kietäväinen et al., 2014). The two fracture zones investigated here have previously been described in detail (Purkamo et al., 2016). In brief, the concentration of sulfate detected at 180 and 500 m was 1.5 and 1.0 mg l^-1^, respectively, in the original fracture water. At 500 m depth, the gas to water ratio was 0.7 and methane contributed with 22 mmol l^-1^ gas (Kietäväinen et al., 2013). The methane concentration at 180 m is not known.

### Sample Collection

Deep subsurface fracture fluids were collected in October 2010 (500 m fracture) and June 2012 (180 m fracture). The fracture zones in question were isolated from the rest of the drillhole by inflatable packers (Ahonen et al., 2011). The sampling was conducted as described previously (Purkamo et al., 2013, 2016). The isolated fracture zones were purged for 21–42 days, depending on the water yield of the correspondent fracture. Care was taken to ensure that the pumping rate did not exceed the rate of inflow from the fracture zone. Temperature, pH, electrical conductivity (*E*_c_), concentration of O_2_ and redox potential (*E*_h_) of the pumped fluid was continuously monitored in a flow-through cell and have been reported previously (Purkamo et al., 2016).

The fluid from both fracture zones was collected in the field into sterile, acid-washed 2000 ml glass bottles (Schott, Germany) in an anaerobic chamber (MBraun, Germany). Biomass for RNA and DNA extraction describing the original microbial communities was collected from two duplicate 500 ml samples for each nucleic acid fraction on 0.22 μm filters as described earlier (Rajala et al., 2015). In order to preserve the microbial RNA, the sample processing time was kept short and filters were frozen on dry ice immediately after sample collection and maintained at -80°C until nucleic acid isolation.

### Induction of Microbial Activity by Methane or Methanol

The microbial communities’ response to methane (final concentration 90 mM) (referred here as CH_4_) and methanol (final concentration 0.2 mM) (referred here as MetOH) was examined by introducing substrates to aliquots of sample fluid (500 ml). Sulfate (final concentration 0.375 mM, referred here as SO_4_) was added in addition of CH_4_ and MetOH to study if addition of possible electron acceptor changes microbial communities interactions in methane/methanol metabolisms. Sample water was divided in to sets of three parallel 500 ml subsamples to be treated with four different substrate combinations. MetOH and SO_4_ were added to samples in an anaerobic glove box and CH_4_ was added through the air-tight rubber cap with N_2_-flushed syringe and needle. Methanol and sulfate were made anoxic prior to use by sterile N_2_ gas flush for 30 min. After the substrate addition, the samples were incubated for 2 h at 14°C in a shaker (45 rpm). The biomass from the incubated samples was collected on 0.22 μm filters. The filters were cut from the filtration funnels with a sterile scalpel and were immediately frozen on dry ice and maintained at -80°C until RNA isolation.

### Nucleic Acid Extraction

DNA was extracted from the filters containing the microbial biomass from the original fracture water (baseline situation) using the PowerWater DNA Isolation kit (MoBio Laboratories, Inc., Carlsbad, CA, USA) in accordance with the manufacturer’s protocol. RNA from both original fracture water and activated samples was extracted from filter membranes using the PowerWater RNA Isolation kit (MoBio Laboratories, Inc., Carlsbad, CA, USA), including DNase, in accordance with the manufacturer’s protocol. Negative reagent controls for nucleic acid extractions were included in each extraction.

### Reverse Transcription

The presence of residual DNA in the RNA extracts was excluded by running a PCR with bacterial universal primers U968f and U1401r (Nübel et al., 1996) using the RNA extract as template. No PCR product was obtained, and it was thus assumed that the RNA extracts did not contain residual DNA and they were submitted to cDNA synthesis. The extracted RNA was converted to cDNA by reverse transcriptase-PCR using the Superscript III First Strand Synthesis SuperMix (Invitrogen, Carlsbad, CA, USA) as described previously (Rajala et al., 2015).

### Amplicon Libraries and Sequencing

The amplification libraries for high throughput sequencing on the Ion Torrent PGM platform (Thermo Fisher Scientific) were prepared by PCR from two replicate DNA samples. Bacterial 16S rRNA genes were amplified with primers S-D-Bact-0341-b-S-17/S-D-Bact-0785-a-A-21 (Herlemann et al., 2011), targeting the variable region V3-V4 of the 16S rRNA gene, archaeal 16S rRNA genes with primers S-D-Arch-0349-a-S-17/S-D-Arch-0787-a-A-20 (Klindworth et al., 2013), targeting the V4 region of the gene. PCR amplification was performed in parallel 25 μl reactions for every sample containing 1 × MyTaq^TM^ Red Mix (Bioline, London, UK), 20 pmol of each primer, up to 25 μl nuclease-free water (Sigma, St. Louis, MO, USA) and 2 μl of template. The PCR program consisted of an initial denaturation step at 95°C for 3 min, 35 cycles for bacteria and 40 cycles for archaea of 15 s at 95 °C, 15 s at 50°C and 15 s at 72°C. A final elongation step of 30 s was performed at 72°C. Correct size of the PCR products was verified with agarose gel electrophoresis. Amplicon libraries were sent to Bioser, University of Oulu (Finland) for sequencing on the Ion Torrent PGM equipment (Thermo Fisher Scientific), and amplicons were purified and size selected to include only 400–600 bp amplicons on a Shimadzu capillary electrophoresis (Kyoto, Japan) prior to sequencing.

### Sequence Analysis

The sequence reads obtained from Ion Torrent sequencing were subjected to quality control using the QIIME-software Version 1.9 (Caporaso et al., 2010) using a minimum quality score of 20, minimum and maximum sequence length of 200 and 600 bp, respectively, maximum primer mismatch of two nucleotides (nt) and maximum homopolymer stretches of eight nt. Adapters, barcodes and primers were removed from the sequence reads, and chimeric sequence reads were removed from the dataset with the USEARCH-algorithm (Edgar, 2010) by *de novo* detection and through similarity searches against the Greengenes reference dataset (Version gg_13_5) (DeSantis et al., 2006) with bacterial and archaeal sequences. OTUs were picked at 97% sequence homology against the Greengenes database, and *de novo* OTUs were picked from a randomly subsampled sequence subset that failed the closed-reference OTU-picking stage. Singleton OTUs, i.e., OTUs that were represented by a single sequence, were filtered from the dataset. Taxonomy from domain to species level was assigned to OTUs via representative OTU sequences with the Ribosomal Database Project (RDP) classifier algorithm at a minimum of 80% confidence (Wang et al., 2007). Sequences that did not get any taxonomic assignments were removed from the data sets. Alpha diversity calculations were performed on the absolute OUT abundance data as well as on data normalized to 1000 sequence reads per sample for better comparison between samples. Samples with less than 1000 sequence reads were not normalized.

The sequences were deposited in the European Nucleotide Archive (ENA^1^) under accession number PRJEB18131.

### Statistical Analyses and Data Visualization

Alpha-diversity measures (observed OTUs, Chao1 OTU richness and Shannon diversity index) were calculated based on the OTU abundance data outputted by QIIME using the Phyloseq package in R (R Development Core Team, 2013; McMurdie and Holmes, 2015) and visualized using ggplot2. The similarity of the archaeal and bacterial communities between the different treatments was tested by principal coordinate analysis (PCoA) using the Phyloseq package in R. The analysis was performed using the OTU abundance data outputted by QIIME. The Bray–Curtis distance model was used for both analyses. Eigen values for the variance explained by the PCoA dimensions were calculated on 999 permutations using vegan (Oksanen et al., 2016) in R. An UPGMA tree clustering the samples according to similarity in the OTU profiles and a heatmap was calculated using the 1000 most abundant OTUs with the Bray–Curtis similarity model using phyloseq in R. Statistically significant differences in number of observed OTUs, Chao1-estimated richness and Shannon diversity between treatment types (original DNA and RNA, amendments) and between the different depths was analyzed with one-way ANOVA, Tukey’s pairwise test, Kruskal–Wallis and Mann–Whitney pairwise test using the PAST software (Hammer et al., 2001).

### Induction of Respiration and Transcription by Addition of Substrates

The activating effect of methane or methanol on microbial communities was identified by the redox indicating dye 5-cyano-2,3-ditolyl tetrazolium chloride (CTC, Polysciences Inc., Warrington, PA, USA). 50 ml sterile, acid-washed glass serum bottles (Wheaton, NJ, USA) were flushed with sterile N_2_ gas for 30 min, each containing 0.55 ml 50 mM CTC dye, and sealed with sterile butyl rubber stoppers (Bellco Glass Inc., Vineland, NJ, USA) and open top aluminum crimp caps (Sigma, St. Louis, MO, USA). Fracture fluid, after 4 weeks of starvation, was aliquoted (5 ml) under anoxic atmosphere through the butyl rubber stoppers into the sealed infusion bottles using a N_2_-flushed sterile syringe and needle. The bottles were amended with filter-sterilized CH_4_ (9 mM) or anoxic filter-sterilized methanol (0.2 mM) with or without sulfate (anoxic, filter-sterilized, final concentration 0.375 mM).

The samples were incubated together with the CTC dye for 6 h, according to the manufacturers recommendations, at +14°C on a shaker (45 rpm), after which 0.5 ml glycerol-TE buffer was added to the samples for preservation at -80°C according to the protocol by the Single Cell Genomics Center^2^. Briefly the samples were divided into 1 ml aliquots in sterile cryo-tubes and immediately frozen in liquid N_2_ and stored at -80°C. A non-dyed reference sample was prepared and preserved in the same way as the dyed samples.

The CTC-dyed and un-dyed fracture fluid aliquots were carefully thawed on ice prior to screening by flow cytometry (BD FACSaria flow cytometer, Becton Dickinson, Franklin Lakes, NJ, USA). Samples were injected into a sterile phosphate buffered saline (PBS) flow stream and fluorescence was detected using a 655 nm long pass and 675/20 nm band pass filters. For excitation of CTC, an argon laser was used (488 nm). To detect the active respiratory cells, simultaneous measurements of forward light scatter (relative size), side light scatter (cell granularity), and CTC fluorescence emission were used, by setting the PMT voltage to 250, 250, and 500 volts, respectively. The side scatter threshold was set to 2000. To investigate the concentration of respiratory active cells, events were acquired over a time of 30 s with a flow rate of 10 μl min^-1^ from each sample. The fluorescence signal was plotted to the side scatter and analyzed with the BD FACSDiva^TM^ 5.1 software (Becton Dickinson, Franklin Lakes, NJ, USA). The CTC positive cells were gated comparing the fluorescence intensities of the dyed to the un-dyed fracture fluid sample.

For comparison total number of cells was determined by staining with 4’,6-diamidino-2-phenylindole (DAPI) and microscopying as described by Purkamo et al. (2013) and Rajala et al. (2015).

## Results

### Active Population

**FIGURE 1:**
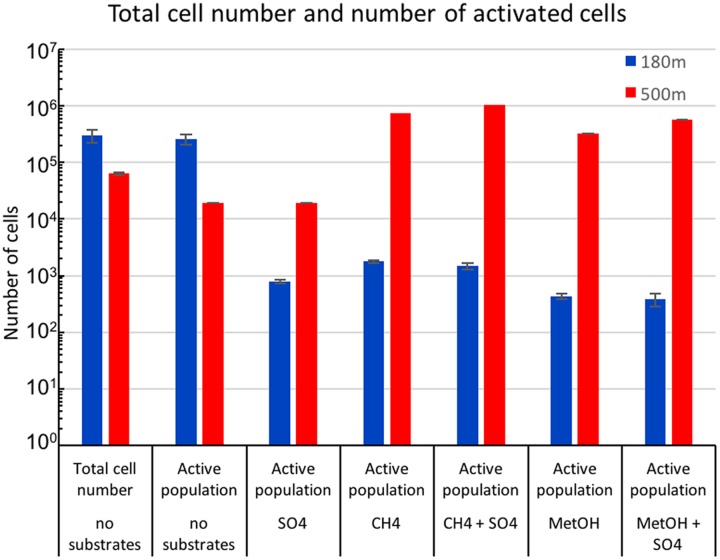
**Mean concentrations of total microbial cells from untreated fracture waters detected with DAPI staining combined to epifluorescence microscopy and active microbial population from untreated and substrate amended fracture water detected by CTC staining combined to flow cytometry**.

The total number of microbial cells in the fracture fluid at the time of sampling was evaluated by counting DAPI stained cells using epifluorescence microscopy (Rajala et al., 2015). In addition, the proportion of respiratorily active cells was determined by staining using the redox sensitive dye, CTC, and counting the cells using flow cytometry. The number of microbial cells was 3 × 10^5^ ml^-1^, of which 87% were respiratory active at the depth of 180 m. At 500 m depth the number of cells was 6 × 10^4^ ml^-1^, of which only 0.3% were active (**Figure 1**). The microbial population from 500 m depth responded to the addition of methane and methanol by increasing metabolic activity. The largest increase in metabolic activity (16.3%) was seen in CH_4_+SO_4_ amended sample (**Figure 1**). Sulfate alone did not affect the metabolic activity of the microbial community of the 500 m depth and methane amendment caused slightly lower increase in the activity (11.6%). Sulfate combined to methanol also increased the activity (9%) slightly more than methanol alone (5.2%). The microbial community from the 180 m depth where the majority of microbial community was respiratory active did not respond in the same way to the amendment of methane or methanol substrates or sulfate as the community at 500 m but the activity of community remained low (**Figure 1**).

### Bacterial Community

**FIGURE 2:**
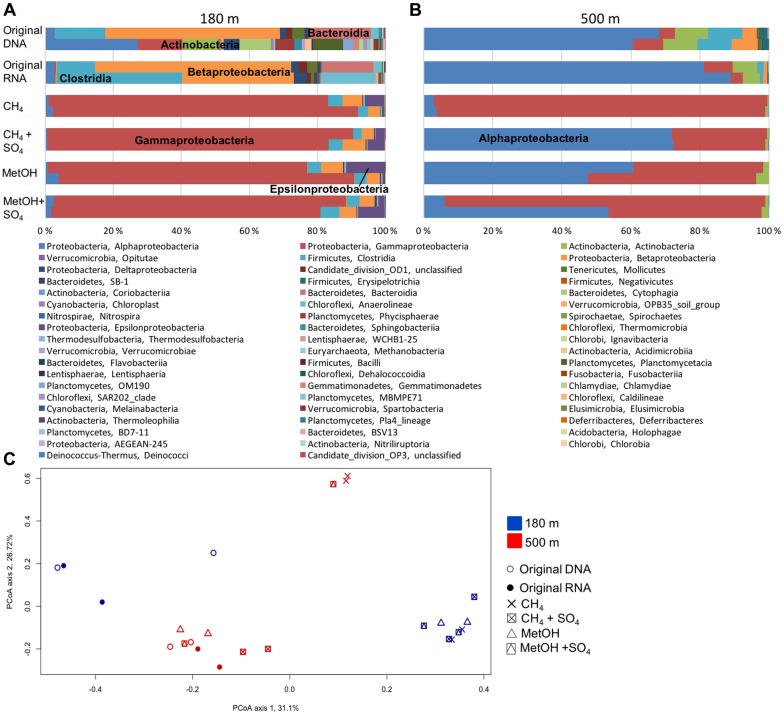
**Relative abundance of bacterial community at (A)** 180 m and **(B)** 500 m depths based on the bacterial 16S rRNA obtained from DNA (total community) or RNA (active community). **(C)** Principal coordinate analysis (PCoA) on all the OTU profiles on the Bray–Curtis distance model for the bacterial communities. PCoA axis 1 explains 31.1% of the variation (eigen value of 999 permutations 1.87) and PCoA axis 2 explains 26.7% of the variation (eigen value of 999 permutations 1.61).

Proteobacteria accounted for the majority of the bacterial communities at both 180 and 500 m depths, but substrate addition changed the 16S rRNA profile at both depths. At the 180 m depth the original fracture water community (DNA and RNA) consisted of Betaproteobacteria, Clostridia, Bacteroidia and Anaerolineae (**Figure 2A**). After methane or methanol addition the detected rRNA profile of RNA contained mainly Gammaproteobacteria (mainly genus *Pseudomonas*), with Clostridia and Betaproteobacteria as minor classes (**Figure 2A** and **Supplementary Figure S1**). This indicates that the *Pseudomonas* significantly increased their production of ribosomes as a result of the addition of substrates. There were no major differences between the systems with different substrates added. At the 500 m depth the original community detected from both the DNA and RNA fractions consisted of Alphaproteobacteria, Actinobacteria and Clostridia (**Figure 2B**). However, after addition of substrates the 16S rRNA profile from RNA was dominated by Alphaproteobacteria (resembling mainly *Rhodobacter*, minority *Caulobacter*) and Gammaproteobacteria (resembling *Pseudomonas*) (**Figure 2B** and **Supplementary Figure S1**).

The number of bacterial sequence reads obtained varied between 1116 and 7376 reads per sample with an average number of 3862 bacterial sequence reads per sample (**Table 1**). The number of observed OTUs obtained from the DNA fraction of the bacterial community of the original fracture water at 180 m depth was generally higher (411–735 OTUs) than that observed at 500 m depth (258–328 OTUs) (**Table 1**). The OTU number detected from the RNA fraction from 180 m depth was also higher (200–492 OTUs) than that detected from the fraction at 500 m (124–281 OTUs). The number of OTUs detected in the amended samples was on average 302 (+/- 22.6 standard error of mean SEM) OTUs in the samples from 180 m and 227 (+/- 8.1 SEM) OTUs in the samples from 500 m, which was statistically significantly lower (Tukey’s pairwise *p* < 0.01, Mann–Whitney Bonferroni corrected *p* < 0.05). Taken all observed OTUs into account from the two different depths the average number of OTUs was statistically significantly lower (on average 234 OTUs per sample, SEM +/- 14.5 OTUs) in the samples from 500 m compared to the samples from 180 m (on average 355 OTUs per sample +/- 42.7 SEM), *p* < 0.05 according to Tukey’s pairwise test and Mann–Whitney pairwise test with Bonferroni correction. The estimated Chao1 OTU richness was slightly higher in the samples from 180 m compared to those from 500 m (**Table 1**), but no statistically significant differences between the original DNA or RNA fractions or the amended samples between the two depths were observed. The Shannon diversity index *H’* calculated for the bacterial community of the original fracture water at the 180 m depth was 3.5–5.7 and 3.3–3.7 from the DNA and RNA fractions, respectively. In the substrate amended microcosms the *H’* was between 2.1 and 3.2 in all samples, and the differences were not statistically significant. However, when combining the values obtained from the same treatment of the different depths, the average H’ from the different amended samples (i.e., methane, methane+sulfate, methanol, or methanol+sulfate) were all statistically significantly lower, between 2.35 and 2.88 (*p* < 0.05) compared to the *H’* from the original DNA (average *H’* = 4.2) and original RNA (average *H’* = 3.1) fractions combined from both depths.

**Table 1:** The alpha-diversity measures (observed OTUs, Chao1 OTU richness and Shannon diversity index *H*’) calculated for archaeal and bacterial community, numbers 1 and 2 in the sample names refer to two parallel samples included in analysis.

Depth	Sample	Number of sequences	Number of Observed OTUs	Number of Chao1-estimated OTUs	Shannon diversity index *H’*	^∗^Number of Observed OTUs	^∗^Number of Chao1-estimated OTUs	^∗^Shannon diversity index *H’*
Archaea								
180 m	Original DNA1	3131	47	56	3.1	37	50	3.1
	Original DNA2	5855	36	47	2.0	25	27	2.0
	Original RNA1	7043	45	52	3.0	28	33	2.9
	Original RNA2	3443	26	28	1.7	17	17	1.6
	CH_4_ - 1	2081	22	25	1.4	17	17	1.4
	CH_4_ - 2	1505	26	35	1.5	24	30	1.4
	CH_4_+SO_4_ - 1	310	16	18	1.7	–	–	–
	CH_4_+SO_4_ - 2	2906	22	22	1.2	21	29	1.3
	MetOH - 1	2868	20	21	0.7	17	21	0.8
	MetOH - 2	2345	31	38	2.2	25	29	2.2
	MetOH+SO_4_ - 1	2645	19	19	1.4	16	17	1.4
	MetOH+SO_4_ - 2	2817	21	27	1.2	18	19.5	1.1
500 m	Original DNA1	891	36	66	3.0	–	–	–
	Original DNA2	66	5	6	1.5	–	–	–
	Original RNA1	–	–	–	–	–	–	–
	Original RNA2	131	8	23	1.3	–	–	–
	CH_4_ - 1	1103	25	130	2.3	24	115	2.2
	CH_4_ - 2	255	14	18	1.9	–	–	–
	CH_4_+SO_4_ - 1	1511	24	42	2.5	22	36	2.5
	CH_4_+SO_4_ - 2	2332	20	22	2.0	17	24	2.0
	MetOH - 1	–	–	–	–	–	–	–
	MetOH - 2	2650	58	89	2.9	37	63	2.7
	MetOH+SO_4_ - 1	3441	35	95	2.0	20	27	2.0
	MetOH+SO_4_ - 2	655	23	28	2.8	–	–	–
Bacteria								
180 m	Original DNA1	3102	735	1427	5.7	408	851	7.9
	Original DNA2	3152	411	1075	3.5	194	488	5.0
	Original RNA1	4174	492	1322	3.3	193	661	4.6
	Original RNA2	1683	200	323	3.7	161	335	5.3
	CH_4_ - 1	4143	197	497	2.1	95	179	2.9
	CH_4_ - 2	4482	246	463	2.2	103	230	3.0
	CH_4_+SO_4_ - 1	4591	274	598	2.4	109	310	3.2
	CH_4_+SO_4_ - 2	7376	300	534	2.4	93	254	3.2
	MetOH - 1	5005	329	676	2.3	118	268	3.2
	MetOH - 2	6981	406	767	3.1	138	383	4.4
	MetOH+SO_4_ - 1	4335	343	866	3.2	147	346	4.4
	MetOH+SO_4_ - 2	5042	325	652	2.5	127	313	3.5
500 m	Original DNA1	1771	258	789	3.6	165	565	5.0
	Original DNA2	1662	328	1268	4.0	235	760	5.6
	Original RNA1	3247	281	1101	2.7	123	278	3.9
	Original RNA2	1116	124	399	2.8	110	307	4.0
	CH_4_ - 1	3779	205	604	2.5	82	268	3.5
	CH_4_ - 2	4096	228	425	2.6	95	231	3.6
	CH_4_+SO_4_ - 1	3883	249	579	2.8	110	280	4.0
	CH_4_+SO_4_ - 2	4828	258	826	2.6	89	234	3.6
	MetOH - 1	3116	224	804	3.1	118	256	4.5
	MetOH - 2	4126	248	636	3.0	105	278	4.2

*A dash (–) indicates missing data. ^∗^subsampled to 1000 sequence reads per sample and shown for samples with <1000 reads*.

The PCoA analysis demonstrated that the community profiles from the different samples grouped into several groups. The original DNA and RNA samples from the 180 m depth fell clearly separate from the amended samples (**Figure 2C**). However, of the samples from 500 m, only one MetO+SO_4_ sample and both CH_4_ samples fell into a different group than the original DNA and RNA samples. Of the 500 m samples the two CH_4_-amended samples and one MetOH+SO_4_ sample formed their own cluster separate from the other samples from the 500 m depth (**Figure 2C**). In addition, the samples from the two depths did not mix on the PCoA plot.

**FIGURE 3:**
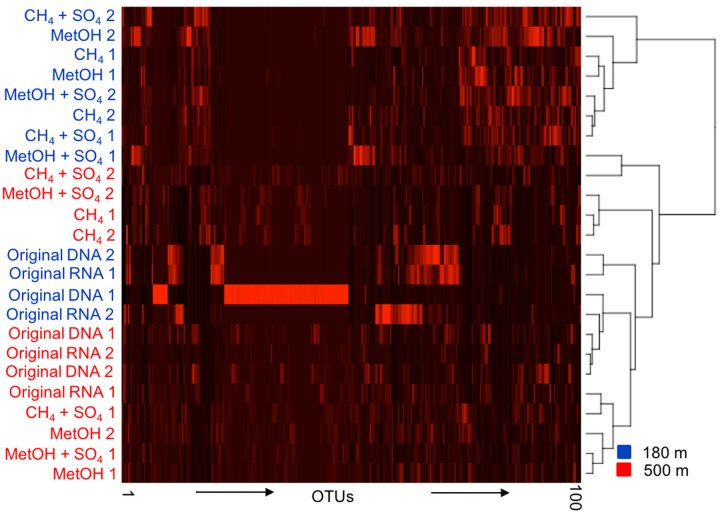
**A UPGMA cladogram clustering the samples based on the 100 most abundant OTUs of the bacterial OTU profile according to the Bray–Curtis distance model**. Blue indicating 180 m depth and red indicating 500 m depth, numbers 1 and 2 refer to two parallel samples included in analysis.

The UPGMA tree and heat map analysis, including the 1000 most abundant OTUs, based of the Bray–Curtis distance model clustered together most of the bacterial communities of the original fracture water determined by both the DNA and RNA fractions from both depths (**Figure 3**). The bacterial communities from the substrate amended microcosms from 180 m clustered together in the heat map, while those from 500 m were split in to two different groups, one containing both CH_4_ amended communities and the other containing both MetOH-amended communities, with CH_4_/MetOH+SO_4_ communities divided between them.

### Archaeal Community

**FIGURE 4:**
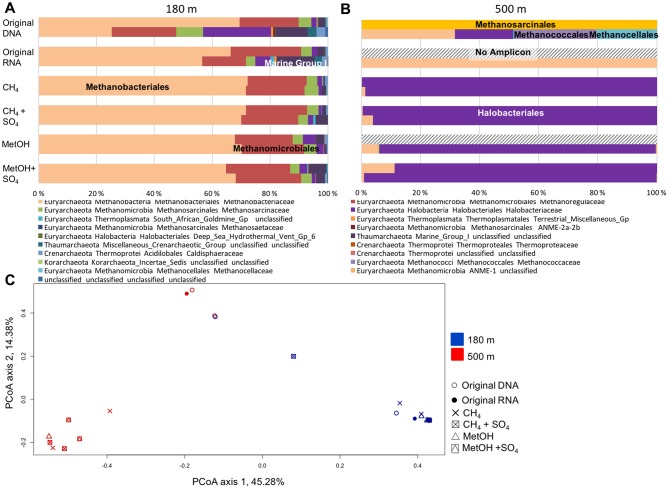
**Relative abundance of archaeal community at (A)** 180 m and **(B)** 500 m depths based on the archaeal 16S rRNA obtained from DNA (total community) or RNA (active community). **(C)** PCoA on all the OTU profiles on the Bray–Curtis distance model for the archaeal communities. PCoA axis 1 explains 45.3% of the variation (eigen value of 999 permutations 3.45) and PCoA axis 2 explains 14.4% of the variation (eigen value of 999 permutations 1.11).

The archaeal communities consisted mostly of the Euryarchaeota at both depths. At the 180 m depth the methane or methanol did not affect the archaeal community composition (**Figure 4A** and **Supplementary Figure S2**). The major order was the Methanobacteriales (**Supplementary Figure S2**). In addition, Methanomicrobiales, Halobacteriales and thaumarcheotal Marine Group I (MGI) were detected (**Supplementary Figure S2**). At the 500 m depth the archaeal community structure changed due the methane or methanol availability (**Figure 4B**). Methanosarcinales, Methanobacteriales, Methanococcales and Methanocellales were detected from the original fracture water samples (**Figure 4B** and **Supplementary Figure S2**). As a result of methane or methanol amendments the rRNA profile of RNA changed and was dominated mainly by Halobacteriaceae that was not detected in active community of untreated fracture water, with a small portion of Methanobacteriaceae, 30% decrease in relative abundance compared to untreated RNA (**Supplementary Figure S2**).

The number of archaeal sequence reads obtained from the different samples varied between 0 and 4896 reads with an average of 1818 sequence reads per sample. The number of observed archaeal OTUs in the original fracture water was 30–79 and 92–100 OTUs in the DNA and RNA fractions, respectively, while in the substrate amended samples the number of OTUs varied between 26–84 archaeal OTUs. The Chao1-estimated number of OTUs in the original fracture water was 60–130 and 149–232 OTUs in the DNA and RNA fractions, respectively and varied between 31–106 in the substrate amended samples. The *H’* calculated for the original fracture water was 1.9–2.1 and 2.5 in the DNA and RNA fraction, respectively and in the substrate amended samples *H’* varied between 1.8–2.5. At 500 m the number of observed and estimated OTUs as well as *H’* was generally lower than those at 180 m. The number of observed OTUs in the original fracture water was 3–24 and 4 in the DNA and RNA fractions, respectively. Nevertheless, the number of observed OTUs increased after substrate amendment to between 12–28. The Chao1-estimated number of OTUs in the original fracture water was 4–38 and 7, DNA and RNA fractions, respectively, and varied between 17–61 after substrate amendment. The *H’* calculated for the original fracture water at 500 m depth 0.3–2.2 and 0.2 for DNA and RNA fractions, respectively. In the substrate amended samples the *H’* varied between 1.3–1.7. Overall, the archaeal community had higher OTU richness and *H’* at 180 m than 500 m depth. Nevertheless, there were no statistically significant differences in the number of detected or Chao1-estimated OTUs or *H’* between the different depths or sample types.

**FIGURE 5:**
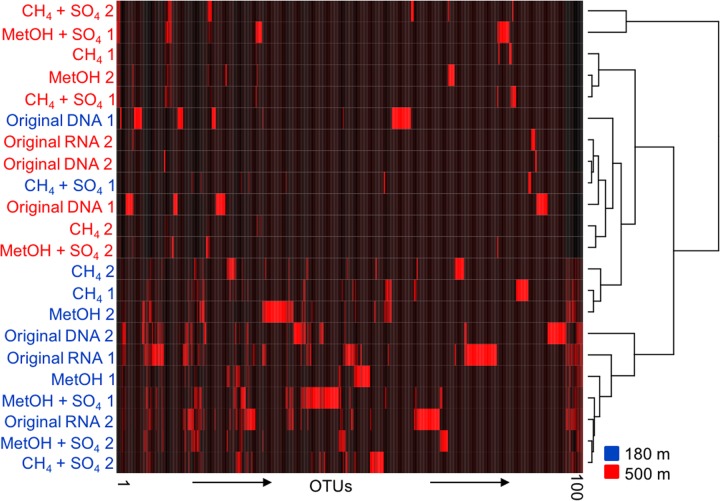
**A UPGMA cladogram clustering the samples based on the 100 biggest OTUs of the archaeal OTU profile according to the Bray–Curtis distance model**. Blue indicating 180 m depth and red indicating 500 m depth, numbers 1 and 2 refer to two parallel samples included in analysis.

At both depths the active archaeal community detected from the RNA fraction in the original fracture water was more diverse than that detected from DNA fractions. The PCoA plotting of the archaeal community showed clear separation between the depths (**Figure 4C**). The archaeal communities from the 180 m fracture zone were similar to the ones detected from the original fracture water and generally clustered together both on the PCoA plot and in the UPGMA tree (**Figures 4C**, **5**). Nevertheless, the archaeal community profile of the substrate amended microcosms from the 500 m fracture zone clustered as a group to the left of the PCoA plot as well as in the UPGMA tree. This division indicates that a different population from that dominating in the fracture water at 500 m depth increased their transcription of ribosomes after substrate amendment allowing for more frequent detection of the ribosomal RNA of this activated population compared to the ribosomes of the dominating taxa.

## Discussion

Microbial communities in deep, nutrient limited subsurface environments of areas with low seismic activity are assumed to express only low metabolic activity when the environmental factors remain stable (Jørgensen and D’Hondt, 2006; Jørgensen, 2011; Hoehler and Jørgensen, 2013; Lever et al., 2015). In the present study the activity of microbial community differed largely between the two studied fracture zones, the communities of more shallow fracture, 180 m depth, being more active (87% of population active) than the communities of deeper fracture zone, 500 m depth, where only 0.3% of community was active. By remaining in dormant state the microorganisms lower their energetic expenditures. In ancient isolated subterranean fracture zones, the fluids and the surrounding rock have developed a state of equilibrium where the fluid does not leach chemical substances from the rock without external interference. Occasionally gas may be released causing a stirring in the fluid bringing a flush of nutrients or electron acceptors from the depths, but in general the deep subsurface environment is deemed stable. Nevertheless, due to land movements, such as faults on a longer geological time scale, or geological constructions, new passages for release of pressure (gas bubbles) and fluid flow may occur, and infiltration from meteoric water sources may increase. Mixing of chemically distinct fluid faces has been shown to increase microbial metabolic activity as well as change the metabolic pattern in microbial communities in deep groundwater (Pedersen, 2013). The effect of environmental changes due to for example above mentioned mechanisms was simulated by adding methane or methanol with or without sulfate to the fracture water samples. The microbial community at the 500 m depth that appeared dormant, gained respiratory activity rapidly when methane became available (11.6–16.3% activity versus only 0.3% activity of the population in untreated fracture water) demonstrating the viability and ability of long-isolated microbial communities to utilize introduced substrates, as reported also earlier (Morono et al., 2011; Pedersen, 2013; Rajala et al., 2015). However, in the shallower fracture zone, 180 m depth, where the ratio of active cells was higher, only a small sub-population was responsive to the methane or methanol amendment. Earlier, Purkamo et al. (2014) suggested that heterotrophic mechanisms such as fermenting could be community supporting strategies at certain depths of Outokumpu deep biosphere. When comparing the activated bacterial population, it was seen that at 180 m depth the *Pseudomonas* genus (Gammaproteobacteria) formed the majority of the activated community, whereas in the original community they were in minority. At 500 m depth Rhodobacterales order (Alphaproteobacteria) were enriched when methane or methanol compounds were available, in addition to *Pseudomonas*. Earlier Rhodobacterales have demonstrated to utilize methanol efficiently in seawater (Sargeant et al., 2016). Purkamo et al. (2016) suggested that the Pseudomonadaceae-related bacteria are part of the core community in deep crystalline bedrock fractures in Outokumpu and some species affiliating to *Pseudomonas* are able to grow on methanol (Riis et al., 2003).

The archaeal community at 180 m did not respond to methane or methanol amendment, whereas the archaeal community profile at 500 m depth changed from different methanogenic taxa to *Halarchaeum* after methane or methanol amendment. However, the amount of archaeal 16S rRNA sequence reads was low in untreated samples, both DNA and RNA. This is no surprise, because the detection of archaea from these two depths is difficult as archaea have been shown to be present at only low concentrations of 6.2 × 10^3^ and 8.6 × 10^1^ 16S rRNA gene copies ml^-1^, while the bacterial 16S rRNA gene concentration reaches 6.3 × 10^6^ and 1.9 × 10^6^ gene copies ml^-1^ at 180 and 500 m, respectively (Purkamo et al., 2016). Halobacterial groups were only detected from the RNA fraction of the original groundwater, but in the DNA fraction they were not abundant enough to be detected. The fact that the *Halarchaeum* was detected in the RNA fraction of the substrate induced microcosms indicates that although their relative abundance in the community is low they are able to increase their production of ribosomes upon activation, which allows them to be detected. Our results are in accordance with Manucharova et al. (2016) who showed that archaea belonging to the Halobacteriaceae family can rapidly reactivate after long-term dormancy. The microbial community activating effect of methane addition has been demonstrated also earlier at different terrestrial deep biosphere site (Pedersen, 2013). The microbial community there responded to methane addition by increasing their metabolic activity but the activated community did not correspond to known anaerobic methane oxidizers, which would be most straight forward explanation and the full mechanisms behind activation remained unclear (Pedersen, 2013).

According to the water chemistry the two fracture zones at 180 and 500 m depth differ from each other greatly and are thought to represent distinctive water types (Kietäväinen et al., 2013). The shallower fracture zone is characterized with higher pH than the other water types in Outokumpu deep drillhole (Kietäväinen et al., 2013). The fracture water at 500 m depth, on the other hand, has been demonstrated to contain the highest amount of dissolved gases of all fracture zones crossing the deep drillhole. Approximately 75% of dissolved gases (22–32 mmol l^-1^) is methane (Kietäväinen et al., 2013). As reviewed by Kietäväinen and Purkamo (2015) the concentration of methane in Outokumpu deep bedrock is high and is in the same range as concentrations detected in the Lupin mine (Canada) and the Beatrix mine (South Africa). The fracture zones are believed to lack hydraulic connections and the exchange of the fracture fluids between the fracture zones is not considered probable (Nyyssönen et al., 2014). The prevalent rock types at these fracture zones are also different. Mica schist-biotite gneiss is the dominating rock type of the fracture at 180 m depth and Chlorite–sericite schist at 500 m depth (Purkamo et al., 2016). Despite the differences in the water chemistry and the rock types, the original microbial communities identified from DNA and RNA fractions obtained directly from the fracture waters from both depths clustered closely together in the UPGMA tree (**Figures 3**, **5**). Purkamo et al. (2016) speculated that these two fracture zones might both be in contact with ophiolite-derived rock types and serpentinization could support microbial communities and explain the similarity of the two communities originating from separate fracture zones. In addition, the differences in the two communities was also seen as generally higher bacterial OTU numbers detected from the fracture water from 180 m compared to that from 500 m. This was also previously reported by Purkamo et al. (2016).

Even though the original microbial communities clustered together, the activity of microbial communities at these two fracture zones was very different. At 180 m 87% of the microbial cells stained active without being induced with substrates while only 0.3% did so at 500 m depth. Dormancy is estimated to be a common strategy of microorganisms in various environments, especially in deep biosphere (Jørgensen, 2011; Lennon and Jones, 2011). The low activity of the microbial community from 500 m depth is well in line with previous research, whereas an activity rate of 87% at 180 m depth might be considered unusually high for deep terrestrial biosphere (Lennon and Jones, 2011). In addition to higher activity, the cell abundance at 180 m depth was 10-fold more numerous than at 500 m depth. However, it is typical that the number of microbial cells decreases as a function of depth (Jägevall et al., 2011; Purkamo et al., 2013; Nyyssönen et al., 2014). The dying of the microbial community with respiratory active dye combined to flow cytometry counting is likely not sensitive enough to detect the microbial community that is viable but respiring at extreme low level in original untreated fracture water samples. How non-sporulating microbial cells, such as those dominating in this study, might exit dormancy and how they might detect permissive environmental conditions is not clear (Dworkin and Shah, 2010; Lennon and Jones, 2011). Dormant cells need to identify growth-promoting conditions, but the wide range of possible conditions makes such a determination challenging (Dworkin and Shah, 2010). This rapid reactivation has only been tested in few studies but some evidence of reactivation within hours has been shown previously (Whitesides and Oliver, 1997).

Our results also support the idea that microorganisms present as minor groups in an environment may play an important role when microbial communities face changes in environmental conditions. These low-abundance groups may represent the so called rare biosphere (Sogin et al., 2006). The rare biosphere does not necessarily need to consist of taxa that generally are hard to find, but they may represent the minority in, for example, the fracture water habitats investigated here. The rare (or low-abundance) taxa may represent a reservoir of genetic diversity that can respond to environmental changes although the metabolic status of these taxa is unclear (Jones and Lennon, 2010). The rare biosphere may be part of the dormant microbial community and as such are thought to serve as an important reservoir to ensure the survival and diversity of a community under stressful environmental situations. These microbes may reactive and multiply following for them beneficial environmental change, a so called seed bank hypothesis (Jones and Lennon, 2010; Lennon and Jones, 2011). It is likely that both of these mechanisms support the response seen in Outokumpu fracture zone communities.

In the Outokumpu deep drillhole water the hydrogen is in isotopic equilibrium, in the system H_2_O-H_2_-CH_4_ at ambient temperatures, which is thought to either indicate equilibration due to long residence time of groundwater or relatively recent *in situ* production of methane (Kietäväinen et al., 2013). In addition, marker genes for methanogenesis have been previously detected throughout the drillhole water column (Nyyssönen et al., 2014; Purkamo et al., 2016).

## Conclusion

The respiratory activity of microbial communities in terrestrial subsurface fracture zones differed largely between fracture zones at 180 and 500 m depth. Despite apparent dormancy at the 500 m the microbial community may be able to reactivate rapidly when suitable conditions arise. The microbial community of the fracture zone at 180 m depth was originally metabolically more active than the community of the fracture zone at 500 m, and only a small sub-population was able to utilize the newly available carbon source. However, the composition of substrate activated microbial communities differed at both depths from the original communities. Our results demonstrate that the minority OTUs of the total community may play an important role when the communities face environmental changes in its living habitat.

## Author Contributions

PR and MB designed the work, performed the samplings, analyses, and interpreted the data. PR and MB wrote the article.

## Conflict of Interest Statement

The authors declare that the research was conducted in the absence of any commercial or financial relationships that could be construed as a potential conflict of interest.

## Footnotes

1 https://www.ebi.ac.uk/

2 https://scgc.bigelow.org/PDFs/Sample_cryopreservation_glyTE.pdf

## Supplementary Material

FIGURE S1 **The composition of Alphaproteobacteria and Pseudomonadales communities**.

FIGURE S2 **The composition of Archaeal, Methanobacteriales and Halobacteriales communities**.
