# Supplementary figures and images for: Reactivation of Deep Subsurface Microbial Community in Response to Methane or Methanol Amendment
Source: Front Microbiol. 2017 Mar 17;8:431. doi: 10.3389/fmicb.2017.00431 (PMC5355647; doi:10.3389/fmicb.2017.00431)

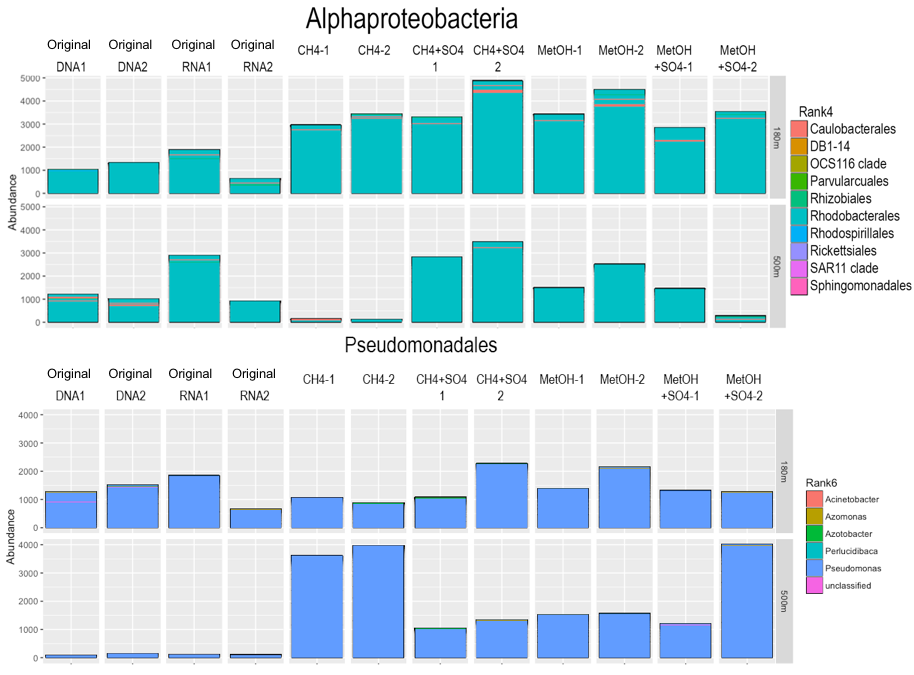

Supplement: FIGURE S1 — The composition of Alphaproteobacteria and Pseudomonadales communities. [file Image_1.TIFF]

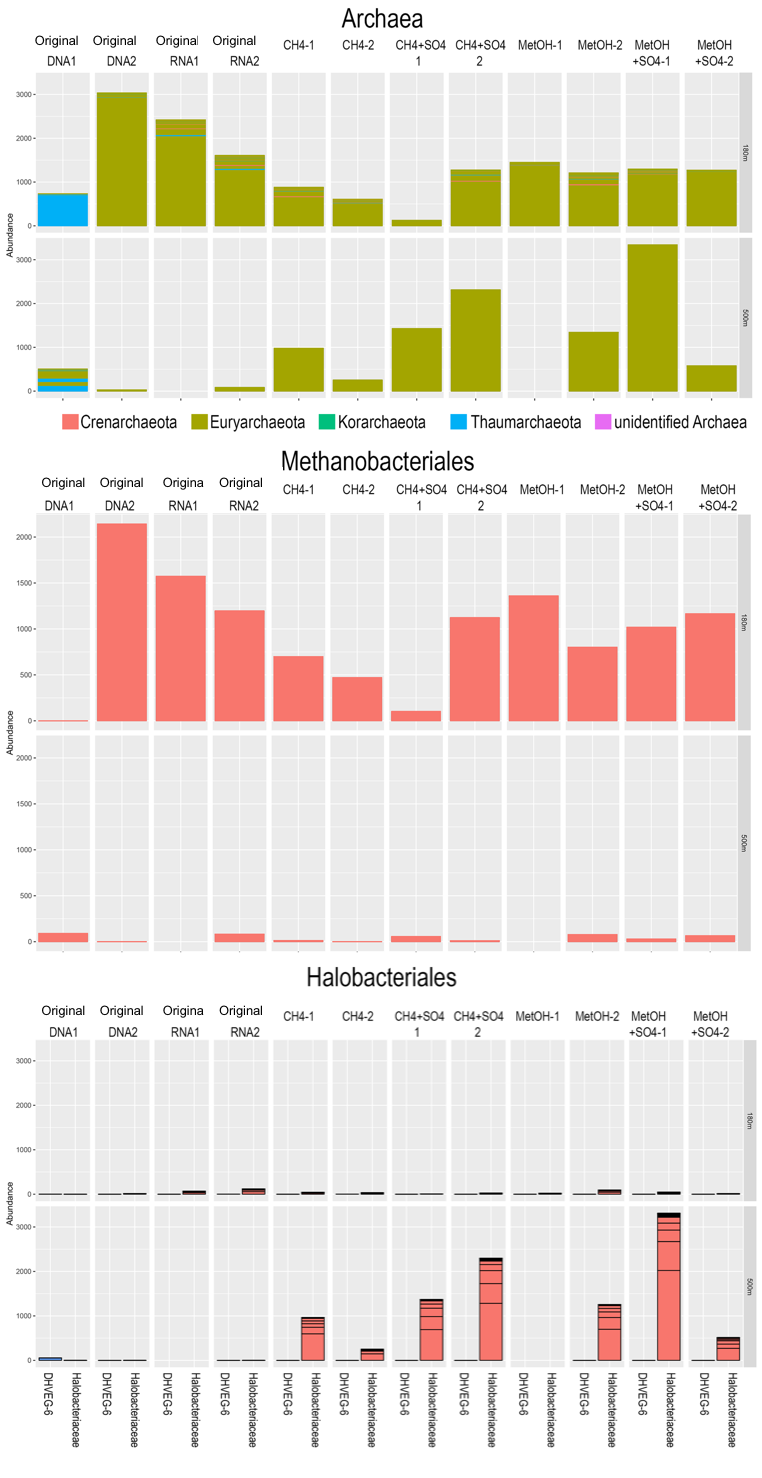

Supplement: FIGURE S2 — The composition of Archaeal, Methanobacteriales and Halobacteriales communities. [file Image_2.TIFF]
